# The effect of repeated stress on KCC2 and NKCC1 immunoreactivity in the hippocampus of female mice

**DOI:** 10.1016/j.dib.2015.12.041

**Published:** 2016-01-07

**Authors:** Takao Tsukahara, Masaaki Masuhara, Haruki Iwai, Takahiro Sonomura, Tomoaki Sato

**Affiliations:** a Department of Applied Pharmacology, Graduate School of Medical and Dental Sciences, Kagoshima University, 8-35-1 Sakuragaoka, Kagoshima, Kagoshima 890-8544, Japan; b Department of Oral Anatomy and Cell Biology, Graduate School of Medical and Dental Sciences, Kagoshima University, 8-35-1 Sakuragaoka, Kagoshima, Kagoshima 890-8544, Japan; c Department of Anatomy II, School of Medicine, Kanazawa Medical University, 1-1 Daigaku, Uchinada, Kahoku, Ishikawa 920-0293, Japan

**Keywords:** KCC2, K^+^–Cl^−^ co-transporter, NKCC1, Na^+^–K^+^–2Cl^−^ co-transporter, pKCC2^ser940^, serine 940 phosphorylated KCC2, RS, repeated stress, (IR), immunoreactive, IHC, immunohistochemistry, DG, dentate gyrus, GC, granular cell, CA3, cornus ammonis 3, CA1, cornus ammonis 1, PFA, paraformaldehyde, PB, phosphate buffer, PBS, phosphate buffered saline, PBS-X, phosphate buffered saline-triton X, RFI, raw fluorescence intensity, KCC2, NKCC1, repeated stress, IHC

## Abstract

K^+^–Cl^−^ co-transporter (KCC2) and Na^+^–K^+^–2Cl^−^ co-transporter (NKCC1) are the main regulators of neuronal intracellular chloride concentration; altered expression patterns of KCC2 and NKCC1 have been reported in several neurodegenerative diseases. In this paper, we show the effect of repeated stress on KCC2, NKCC1, and serine 940 phosphorylated KCC2 (pKCC2^ser940^) immunoreactivity.

The data were obtained from the hippocampus of female mice using single-plane confocal microscopy images. The mean fluorescence intensity of the perisomatic area of neurons, defined as raw fluorescence intensity (RFI) was calculated. Repeated stress (RS) resulted in a decrease in perisomatic area of immunoreactive (IR)-KCC2 and an increase of the IR-NKCC1. In addition, RS decreased perisomatic IR-pKCC2^ser940^, corresponding to that of KCC2. The data in this article support the results of a previous study [1] and provide the details of immunohistological methods. Interpretation of the data in this article can be found in “Repeated stress-induced expression pattern alterations of the hippocampal chloride transporters KCC2 and NKCC1 associated with behavioral abnormalities in female mice” by Tsukahara et al. [1].

**Specifications Table**

**Table:** 

Subject area	Neuroscience
More specific subject area	Neuropsychiatry
Type of data	Single-plane images from confocal microscopy and graphs
How data was acquired	Images were taken with a confocal microscope and analyzed using the Image J software
Data format	Images and graphs
Experimental factors	Repeated stress on mice for 21 days
Experimental features	Images of the KCC2, NKCC1, and pKCC2^ser940^ with raw perisomatic fluorescence intensity
Data source location	Kagoshima, Japan
Data accessibility	The data is in this article and is related to [1]

## 1 Value of the data

• The effects of repeated stress on hippocampal KCC2 and NKCC1 in female mice are shown as single-plane images.
• The RFI of KCC2 and NKCC1 was evaluated in each subregion of the hippocampus.
• We show the immunoreactive image data of pKCC2^ser940^ in the hippocampus, and this is the first report showing pKCC2^ser940^ immunoreactivity in any region of the brain.
• The data show localization based relation of KCC2, NKCC1 and pKCC2^ser940^.

## 2 Data

**Fig. 1:**
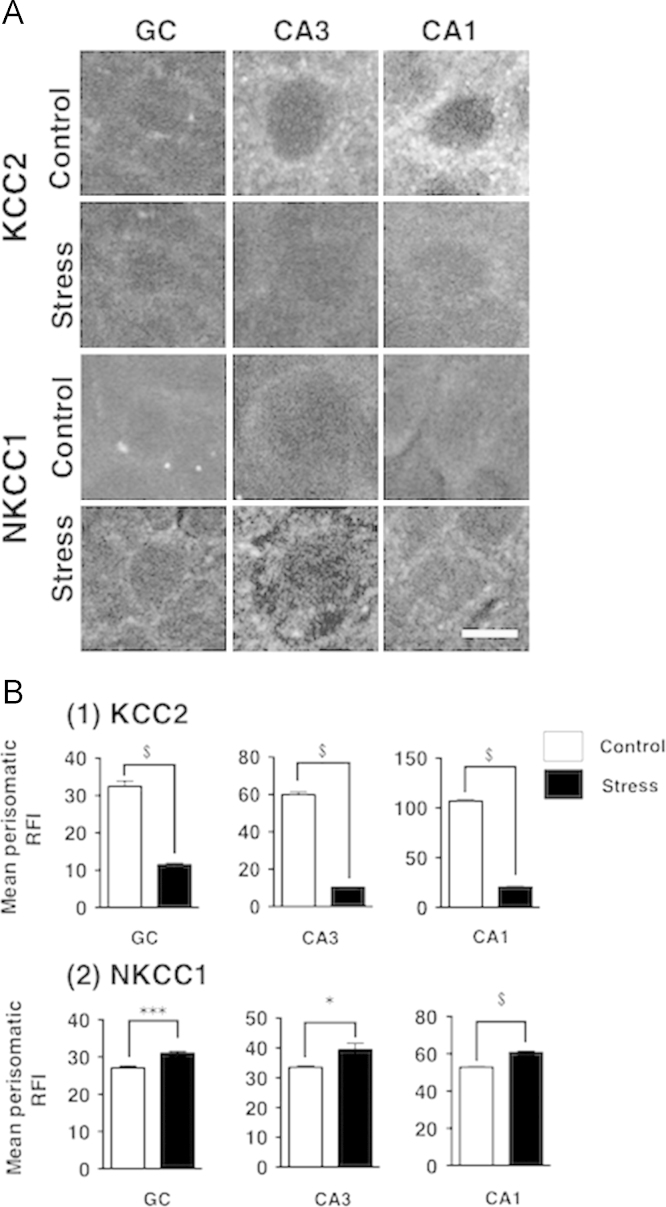
Single plane analyses of the perisomatic KCC2 and NKCC1 expression in the subregions of the hippocampus following RS. The confocal micrographs showing IR-KCC2 and -NKCC1 in the granular cells (GC) in the dentate gyrus and the pyramidal cells in CA3 and CA1 regions of the hippocampus from control and stressed mice. (A) IR-KCC2 and -NKCC1 images; (B) Mean fluorescence intensity of perisomatic KCC2 (1) and NKCC1 (2). Scale bar, 10 µm. *n*=6 and *n*=5 for the control and stressed groups, respectively. Mean perisomatic RFI: mean perisomatic raw fluorescence intensity. *P<0.05, **P<0.01, ***P<0.001, and ^$^*P*<0.0001 vs. controls.

**Fig. 2:**
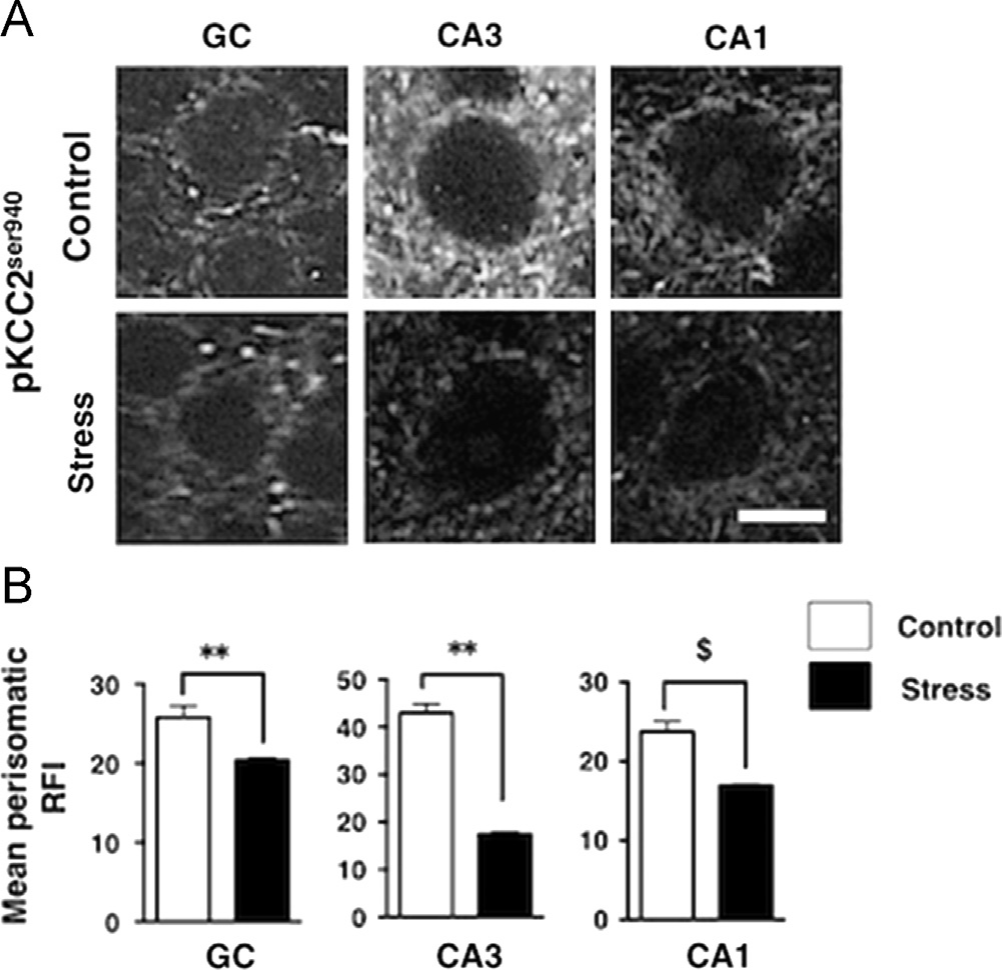
Single plane analyses of pKCC2^ser940^ in the subregions of the hippocampus following RS. The confocal micrographs showing IR-pKCC2^ser940^ in the granular cell (GC) in the dentate gyrus and the pyramidal cells in CA3 and CA1 regions of the hippocampus from control and stressed mice. (A) IR-pKCC2^ser940^ images; (B) Mean fluorescence intensity of perisomatic pKCC2^ser940^. Scale bar, 10 µm. *n*=6 and *n*=5 for the control and stressed groups, respectively. Mean perisomatic RFI: mean perisomatic raw fluorescence intensity. *P<0.05, **P<0.01, ***P<0.001, and ^$^*P*<0.0001 vs. controls.

We investigated the effect of RS on the expression patterns of KCC2 and NKCC1 in the subregions of the hippocampus of female mice, namely granular cells of the dentate gyrus (DG) and pyramidal cells of the CA3 and CA1 subregions. Immunohistochemical staining was performed and images were taken using confocal microscopy. We show the images of immunoreactive (IR)-KCC2 and -NKCC1 expression in each subregion of the hippocampus in control and stressed mice in Fig. 1-A, and the perisomatic RFI of the images is shown in Fig. 1-B. In short, RS decreased perisomatic KCC2 and increased perisomatic NKCC1 in all regions. In the next series of experiments shown in Fig. 2, we focused on pKCC2^ser940^. Images of IR-pKCC2^ser940^ in the hippocampus of control and stress mice are shown in Fig. 2-A. Corresponding to KCC2 data, stressed mice displayed a decrease in the perisomatic RFI of pKCC2^ser940^ in all regions of the hippocampus (Fig. 2-B).

## 3 Experimental design, materials, and methods

### 3.1 Animals

Eleven 8-week-old female C57/BL6J mice were purchased from Kyudo Ltd. (Kumamoto, Japan) and housed in an air conditioned room at a temperature of 24±1 °C with a humidity of 50±10% and under a 12-h light/dark cycle with free access to standard food and water. All experimental procedures were approved by the Animal Research Committee of the Kagoshima University and performed in accordance with the Guidelines of the National Institutes of Health.

### 3.2 Stress induction protocol

Mice were acclimated to their new environment for at least 7 days prior to the experiments (Days 1–7) and were randomly assigned to either the control or the stress group (*n*=6 and *n*=5, respectively). Stress was induced by a forced administration of water (0.1 ml/10 g of body weight) once a day from Day 7 to Day 32 using a corn-shaped tip (diameter of 1.9-mm×4.0-mm) attached to a stainless steel gastric tube [KN-348 (20G-70); Natsume Ltd., Tokyo, Japan]. The gastric tube tip was inserted into the esophagus, and water was slowly administered by force using a 1-ml syringe. During the experimental period, the mice weighed between 17 and 23 g. Because the gastric tube was suitable for mice with a weight of >25 g, the test animals experienced stress during the insertion of the tube and forced water administration.

### 3.3 Immunohistochemistry (IHC)

IHC was conducted as previously described [1]. The mice were perfused and fixed with 4% paraformaldehyde (PFA) in 0.1 M phosphate buffer (PB). Brains were removed and post-fixed in 4% PFA and cryoprotected in 30% sucrose in 0.1 M PB. Following cryoprotection, 30-µm coronal brain sections were cut on a freezing sledge microtome. Tissue sections were quenched with 1% H_2_O_2_ for 1 h, rinsed with phosphate buffered saline (PBS) containing 0.3% (v/v) Triton X-100 (PBS-X), and blocked in 10% normal donkey serum in PBS for 1 h. Tissue sections were then incubated overnight at 4 °C with primary antibodies, namely anti-KCC2, anti-NKCC1, and anti-pKCC2^ser940^ (dilution, 1:1000, 1:2000, and 1:1000 respectively; Millipore, Darmstadt, Germany), rinsed with PBS-X, and incubated with the donkey anti-rabbit biotinylated secondary antibody (1:100; Millipore) for 1 h. Subsequently, sections were rinsed with PBS-X, and incubated in Cy3-conjugated streptavidin (1:200; Sigma) for 1 h, and again rinsed with PBS-X.

### 3.4 Analysis of IHC

IHC analysis was conducted as previously described, with minor modification [2]. Single-plane images of IR-KCC2, -NKCC1, and -pKCC2^ser940^ were obtained using Leica TCS SP5 confocal microscope (objective, 40× and zoom, 5×). All images were taken under the same conditions. Three regions within each area of interest were analyzed on 3–5 sections, with at least one region from each side of the hippocampus. The data used to obtain the RFI were analyzed using the Image J software (NIH, Bethesda, US). The mean perisomatic RFI was quantified in the area surrounding the cell body in individual neurons by using image J. Subsequently, mean values were summarized using Image J. The sum values were divided by the number of analyzed neurons. The mean values were calculated from *n*=3–5 sections, and the mean of control (*n*=6) and stress mice (*n*=5) were reported.

### 3.5 Statistical analysis

All data are expressed as means±standard error of the mean (SEM). The two-tailed Student׳s *t*-test was used for all statistical analyses using GraphPad Prism^®^ 6 software (GraphPad Software Inc., San Diego, CA, USA). Statistical significance was defined as *P*<0.05.

## Conflict of interest

None.

## References

[1] Tsukahara T., Masuhara M., Iwai H., Sonomura T., Sato T. (2015). Repeated stress-induced expression pattern alterations of the hippocampal chloride transporters KCC2 and NKCC1 associated with behavioral abnormalities in female mice. Biochem. Biophys. Res. Commun..

[2] Babaev O., Botta P., Meyer E., Muller C., Ehrenreich H., Brose N., Luthi A., Krueger-Burg D. (2016). Neuroligin 2 deletion alters inhibitory synapse function and anxiety-associated neuronal activation in the amygdala. Neuropharmacology.

